# Association between Age and the 7 Repeat Allele of the Dopamine D4 Receptor Gene

**DOI:** 10.1371/journal.pone.0167753

**Published:** 2016-12-19

**Authors:** Anna Szekely, Eszter Kotyuk, Julianna Bircher, Andrea Vereczkei, David A. Balota, Maria Sasvari-Szekely, Zsolt Ronai

**Affiliations:** 1 Institute of Psychology, Eötvös Loránd University, Budapest, Hungary; 2 Postdoctoral Research Program, Hungarian Academy of Sciences, Budapest, Hungary; 3 Doctoral School of Psychology, Eötvös Loránd University, Budapest, Hungary; 4 Department of Medical Chemistry, Molecular Biology and Pathobiochemistry, Semmelweis University, Budapest, Hungary; 5 Department of Psychology, Washington University in St. Louis, United States of America; George Washington University School of Medicine and Health Sciences, UNITED STATES

## Abstract

Longevity is in part (25%) inherited, and genetic studies aim to uncover allelic variants that play an important role in prolonging life span. Results to date confirm only a few gene variants associated with longevity, while others show inconsistent results. However, GWAS studies concentrate on single nucleotide polymorphisms, and there are only a handful of studies investigating variable number of tandem repeat variations related to longevity. Recently, Grady and colleagues (2013) reported a remarkable (66%) accumulation of those carrying the 7 repeat allele of the dopamine D4 receptor gene in a large population of 90–109 years old Californian centenarians, as compared to an ancestry-matched young population. In the present study we demonstrate the same association using continuous age groups in an 18–97 years old Caucasian sample (N = 1801, p = 0.007). We found a continuous pattern of increase from 18–75, however frequency of allele 7 carriers decreased in our oldest age groups. Possible role of gene-environment interaction effects driven by historical events are discussed. In accordance with previous findings, we observed association preferentially in females (p = 0.003). Our results underlie the importance of investigating non-disease related genetic variants as inherited components of longevity, and confirm, that the 7-repeat allele of the dopamine D4 receptor gene is a longevity enabling genetic factor, accumulating in the elderly female population.

## Introduction

Age at death in adulthood has a heritability of approximately 25% [1]. According to a recent review of genome-wide association studies (GWAS) APOE and FOXO3A gene variants are associated with longevity [2]. Although association of other genetic polymorphisms did not reach the level of genome wide significance, identified pathways and genetic signatures have been shown to be important in longevity [2]. Inheritance of long life span seems to be rather complex, with modest individual genetic effects, along with significant gene–environment interactions.

Based on a study of exceptional longevity, genetic factors seem to be even more important where familial clustering of extreme old age is robust [3]. These individuals might lack some of the risk factors related to various diseases, and at the same time carry protective genetic variations against basic mechanisms of age-related illnesses, also referred to as ‘longevity enabling genes’ [4]. Association between APOE ε4 and longevity for example might be mediated by Alzheimer’s disease; healthy older individuals are less likely to carry the APOE ε4 gene variant, thus they have a smaller risk for Alzheimer’s disease, which further prolongs their life expectancies [5]. On the other hand, a recent GWAS [6] did not show any significant difference in the rate of disease-associated genetic variants in centenarians. Based on a recent review [7] related to the longitudinal New England Centenarian study, GWAS of the oldest old did not reveal statistically significant single gene associations either. Moreover, there was no decline in the frequency of disease-associated genetic variants carried by centenarians as compared to the general population [7]. In addition, A GWAS study of a small sample of the world’s oldest people (17 people over 110 years, 13 of them females) concluded that there is no evidence for enrichment of genetic variants in female Caucasian supercentenarians compared to controls [8].

In contrast to the above studies, a GWAS study proposed specific ‘genetic signatures’ (19 clusters characterized by combinations of certain SNP genotypes) to be correlated with the prevalence and age of onset of age-associated diseases, such as dementia or cardiovascular diseases [9]. Also, a potential negative effect of certain complement C4 haplotypes on health and survival was shown in a Hungarian study by Kramer and colleagues [10], which was more recently replicated by Arason and colleagues [11]. This might be due to non-specific disease susceptibility presumably acting through insufficient immune response [12]. It is also possible, that some genes exert their influence on longevity through personality traits, such as conscientiousness and some of its facets (responsibility, self-control and traditionalism) which have been shown to be significant predictors of extended life-span [13].

It is important to note that due to technical reasons GWAS and SNP studies on longevity have not investigated any variable number of tandem repeat variations (VNTR) in association with longevity. In contrast, Grady and colleagues [14] proposed that a specific VNTR variant, the 7 repeat allele of the dopamine D4 receptor gene (*DRD4*), could be an important factor in extreme longevity, because it plays a major role in the brain’s dopaminergic functioning. According to their findings, surviving participants of a 30-year-old population-based health survey (N = 310, age range 90–109, mean age: 95.2 years) possessed a 66% higher rate of 7 repeat allele carriers as compared to that of an ancestry-matched young population (N = 2902, age range 7–45). In addition, Grady and colleagues found that this association was far more pronounced in females (there were 39.3% allele 7 carriers in the old vs 21.9% in the young population) as compared to males (29.7% in the old vs. 21.9 in the young population). They also reported supporting evidence from animal studies of this gene: The *DRD4* knock-out mice lived 7–9.7% shorter and showed reduced spontaneous locomotor activity, as compared to those with functional *DRD4* genes. Also, while the wild type mice showed clear beneficial effects of an enriched environment on lifespan, the *DRD4* knock-out mice did not a show lifespan increase when reared in an enriched environment.

The *DRD4* is a crucial component in driving behavioral responses to the environment and it has been hypothesized to modulate behavior through reward circuitry, motivation, and attentional mechanisms, which sustain effort focused on a certain behavior [15–17]. The 48 base pair sequence in the third exon of the *DRD4* gene can be repeated from 2 to 11 times [18] and this polymorphism is often referred to as the VNTR in the *DRD4* gene (*DRD4* VNTR). The most common number of repeats is 4 (global mean allele frequency is 64.3%), the second most frequent is 7 (global mean allele frequency is 20.6%), but the frequency of these alleles differ among populations [19].

The DRD4 gene has been associated to various disease-related characteristics; Genopedia [20] lists 180 disease terms associated with the *DRD4* gene. Among various polymorphic sites of the *DRD4* gene, perhaps associations with the *DRD4* VNTR is the most controversial: The 7 repeat form was referred to as the”adventure gene” because of its associations with novelty seeking (e.g. [21], opioid-dependence [22] and attention deficit, hyperactivity disorder [23, 24]. Meta analyses of later studies concluded no clear association for novelty seeking [17, 25, 26] or addiction [27], however, association of the *DRD4* VNTR and ADHD has been consistently replicated [28, 29]. Our laboratory demonstrated that the presence of the 7 repeat allele associates with lower self-reported persistence [30], slower reaction time in cognitive tasks [31] and lower trait impulsivity [32].

According to evidence from resequencing *DRD4* VNTR alleles representing a word-wide population this genetic variant originated as a rare mutational event, which increased in human populations by positive selection [33]. The only existing human study by Grady and colleagues [14] to date related to variations in the *DRD4* VNTR and lifespan compared only extremely old and very young individuals. Hence, systematic comparison of *DRD4* VNTR frequencies in various age groups have not yet been carried out. In fact, initial association result of the *DRD4* VNTR 7 repeat allele and longevity have not yet been replicated to date, which would be reassuring given recent arguments regarding the critical importance of replication in genetic studies [34]. The major goal of the present study was to test association of the *DRD4* VNTR 7 repeat allele and longevity using continuous age groups.

Here we present results of an association study between the 7 repeat allele and longevity using data from a large Hungarian sample from a wide age range. We replicate association results previously demonstrated by Grady and colleagues [14], and also present novel findings using continuous age groups.

## Materials & Methods

Caucasian (Hungarian) subjects participated in different studies of our research group on a voluntary basis. Study protocols were designed in accordance with guidelines of the Declaration of Helsinki, and were approved by the Scientific and Research Ethics Committee of the Medical Research Council (ETT TUKEB), approval number: ad.4514-0/2010-1018EKU(294/PI/10). All participants provided written informed consent at the beginning of the studies. Non-invasive buccal samples and information on age and sex were used in the present study. Participants were recruited from educational facilities (the Institute of Psychology, Eötvös Loránd University and two law enforcement institutions in the Budapest area); retirement homes in the Budapest area; and volunteers recruited on different occasions advertising our research (e.g., the annual Researchers’ Night, Budapest). 91% of young participants were university students living or studying in Budapest, the elderly individuals were typically relatives of university staff or students. We also assembled data from two retirement homes in Budapest. Socio-economic status and cognitive abilities of our participants were typically high.

Those with past or present diabetic or psychiatric history were excluded. Further selection criteria for the analyzed sample included valid *DRD4* genotype (call rate was 98%), valid gender and age data (2 participants were excluded), and genetically independent individuals (65 participants were excluded since their relatives already participated). The sample included 1801 adult participants (age range: 18–97, mean = 30.43 ± 17.83 years, 57.9% females).

### DNA preparation and SNP genotyping

After non-invasive DNA sampling of buccal cells [35], genomic DNA was isolated as described earlier [36]. Genotyping of *DRD4* VNTR was performed as described earlier, unbalanced amplification of the longer and shorter PCR products in heterozygotes was avoided by applying dIMP and low DNA concentration as described earlier [37]. Hardy-Weinberg equilibrium [38] was tested based on the three most common genotypes (44, 47 and 77) of the *DRD4* VNTR. No significant deviation from the Hardy-Weinberg equilibrium was found (*p* = 0.315). Measured frequencies of the *DRD4* VNTR alleles are presented in Table 1. As expected, the 4 repeat allele was the most frequent (66.4%), followed by the 7 repeat allele (17.6%), and the 2 repeat allele (10.2%). Frequencies of the other alleles were below 4%. In accordance with other studies from the literature we used the genotype grouping: 7 repeat present vs. 7 repeat absent in association analyses. Data of the present study is publicly available through the NCBI dbGaP data repository: http://www.ncbi.nlm.nih.gov/gap (search by author). The database can also be downloaded from Figshare: https://figshare.com/articles/Szekely2017PLoS_LongevityDatabase_sav/4272677.

**Table 1:** Frequency values of *DRD4* VNTR alleles.

*DRD4* VNTR	N and ratio of alleles
2 repeat	366 (10.2%)
3 repeat	117 (3.2%)
4 repeat	2408 (66.4%)
5 repeat	29 (0.8%)
6 repeat	12 (0.3%)
7 repeat	634 (17.6%)
8 repeat	32 (0.9%)
9 repeat	4 (0.1%)

### Statistical Analysis

Statistical analyses were carried out in SPSS 20.0 for Windows. Association analyses were carried out in a genotype-wise model. Chi-square tests were used for 2x2 distribution analyses, as well as for testing the Hardy-Weinberg equilibrium. The Jonckheere–Terpstra test was applied to assess distribution of genotypes in a contingency table with ordinal categories of more than two age groups [39]. For comparing gender and age differences of the DRD4 association we applied the Mantel-Haenszel Test for 2x2x2 tables. Correction for multiple testing was used to rule out possible false positive associations [40, 41]. Bonferroni correction was utilized with a corrected level of significance of 0.01 as the nominal p value (0.05) was divided by the number of exploratory analyses performed in this paper (0.05/5 = 0.01). A total of five analyses included testing *DRD4* 7 repeat genotype frequencies in eight age categories; young vs old; males vs females; males and female samples grouped as young vs old.

## Results

### Increased *DRD4* 7 repeat frequency with age in healthy adults

**Fig 1:**
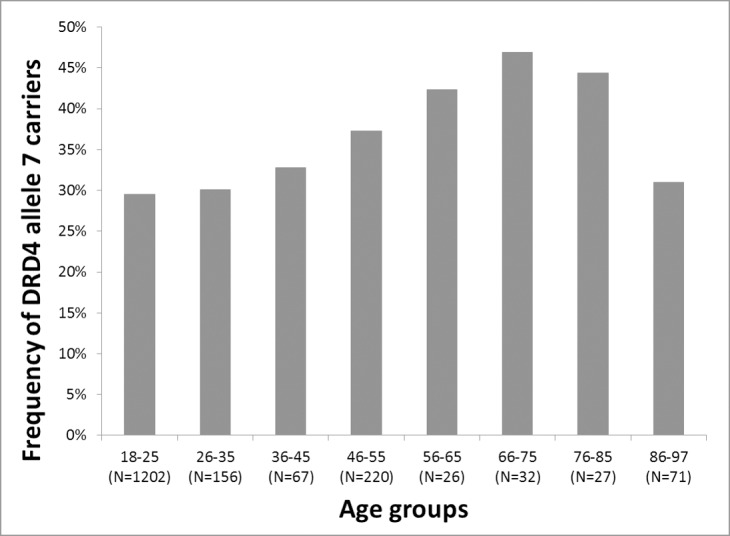
*DRD4* 7 repeat allele frequency of continuous age groups

To replicate and further explore association results previously demonstrated by Grady and colleagues [14] we assessed *DRD4* VNTR data from a large Hungarian sample from a wide age range (18–97). Approximately 10-year age range groups were defined to represent a continuum with at least 25 subjects in each category. The first age group ranged from 18–25 years, then each, with a 10-year range, and finally (at the oldest part of our age spectrum) we applied a 86–97 age range. Frequency of allele 7 carriers in the eight age groups are presented in Fig 1. The independent samples Jonckheere–Terpstra test for ordered alternatives showed a significant increase of those carrying the 7-repeat allele in these age groups (p = 0.007).

According to Fig 1 ratio of 7 repeat allele carriers showed a minor increase, ranging from 29.5% to 32.8% in the three (18–45 years old) younger age groups. However, ratio of 7 repeat carriers gradually increased in the older age groups until age 75 (it was 37.3% in the 46–55 years old, 42.3% in the 56–65 years old and 46.9% in the 66–75 years old subgroups). In the 76–85 age group allele 7 ratio was 44.4%. Interestingly, in the oldest old (86–97 years old) age group of our study the frequency of allele 7 carriers dropped intensively (to 31%). Based on the above frequency results we used dichotomous “young” and “old” age groups with a cutoff point at 45 years in further analyses of gender differences.

### Gender differences in the age related *DRD4* allele 7 frequency changes

Since Grady and colleagues [14] suggested that gender differences exist in the longevity-related increase of the *DRD4* 7 repeat allele frequency, we investigated stratification of those carrying the *DRD4* 7 repeat allele with age in male and female subgroups (data presented in Table 2).

**Table 2:** *DRD4* allele 7 frequency changes with age and gender.

		*DRD4* VNTR genotypes		
		7 absent	7 present	Total
Males	Young (18–45)	430 (69.8%)	186 (30.2%)	616
	Old (46–97)	93 (65.5%)	49 (34.5%)	142
	*Total*	*523 (69*.*0%)*	*235 (31*.*0%)*	*758*
Females	Young (18–45)	571 (70.6%)	238 (29.4%)	809
	Old (46–97)	141 (60.3%)	93 (39.7%)	234
	*Total*	*712 (68*.*3%)*	*331 (31*.*7%)*	*1043*
Total	Young (18–45)	1001 (70.2%)	424 (29.8%)	1425
	Old (46–97)	234 (62.2%)	142 (37.8%)	376
	*Total*	*1235 (68*.*6%)*	*566 (31*.*4%)*	*1801*

First we assessed if allele 7 carriers were over-represented in either sex regardless of age. Presence of the 7 repeat allele was similar in males (31.0%) and females (31.7%). Results of the 2x2 Chi square test was not significant. Then we tested if ratio of allele 7 differed significantly in the young vs. old groups. Among 18–45 year old young participants only 29.8% carried the 7 repeat allele, whereas this ratio was 37.8% for those between 46 and 97 years of age. The 2x2 test showed a significant difference of genotype distribution with age (Chi square = 8.861, df = 1, p = 0.003).

Finally, we conducted an association analysis testing the longevity hypothesis for the male and female subgroups. The Mantel-Haenszel test for 2x2x2 tables showed that association between the *DRD4* 7 repeat allele and age was specific for females (Chi square = 8.411, df = 1, p = 0.004). Presence of allele 7 increased significantly from 29.4% in the young female group, to 39.7% in the old female group (Chi-square = 8.931, df = 1, p = 0.003). The increase was much smaller and was not significant in males: presence of *DRD4* allele 7 was 30.2% in the young and 34.5% in the old group.

P values for all significant results described in the results section were well below 0.01, thus remained significant after the stringent Bonferroni correction.

## Discussion

In the present study we analyzed association of the *DRD4* VNTR with longevity. Association analyses of continuous age groups using genotype data from 1801 Caucasian participants from 18 to 97 years of age showed a significant increase of allele 7 carriers with age (p = 0.007). This result is in line with recent findings of Grady and colleagues [14], who reported a large (66%) increase in frequency of the 7-repeat allele carriers in 90–109 years old Californian centenarians, as compared to an ancestry-matched young population. To our knowledge there are no other reports of this remarkable association, and the present study is the first attempt to investigate association of the *DRD4* VNTR using detailed age ranges (Fig 1). Interestingly, from age 18 to 75 ratio of those carrying the 7-repeat allele increased progressively from 29.5% to 46.9% in the tested age groups, however, in the older age groups the proportion of allele 7 carriers dropped intensively (44.4% in those between 76–85 years and 31% in the 86–97 age group). This “drop” might be due to the relatively small sample size of the age groups, but might also point to the fact that relative importance of environmental, genetic and stochastic determinants of survival vary with age [4]. For example, if one makes it past the more sensitive age range that is typically influenced by a genetic variant, then this affords a marker for the opposite effect for the older age group. Such an effect has been reported for the APOE 4 [42]. Robust environmental factors driven by historical events may also play an important role in enabling gene-environment interaction effects, resulting decrease of a specific allelic variant within a specific age range. The 76+ participants of our Hungarian sample lived through World War II., when over a million Hungarian lives were lost. Moreover, many of them left in 1956, when the revolution erupted in Hungary and people fled the country due to political reasons [43]. 100.000 from the total of over 150.000 emigrants left from those who are over 75 today [44]. Those who left are missing from those age groups that show an intense decrease of allele 7 in our sample. Evidence from the literature on genetic predisposition of migration patterns based on data from over 2000 individuals from 39 populations around the world show a higher proportion of long alleles for DRD4 in migratory populations as compared to sedentary populations [45]. Hence, it is possible that emigration related to political events in 1956 might have been selective for carriers of the 7-repeat allele.

To replicate the effect of the 7 repeat allele on longevity using similar methods to Grady and colleagues [14] we compared allele 7 carriers among 18–25 year olds to the 66–85 year old population, omitting data from those participants who lived through World War II. in their adolescent or young adult years and were young adults or adults in 1956 during the Hungarian revolution. Similarly to earlier findings of Grady and colleagues we observed a large (55%) increase in the ratio of 7 repeat carriers with age. The 29.5% frequency of the 7 repeat allele in 18–25 year old group increased to 45.8% among 66–85 year old group (p = 0.008).

There is no consensus in the literature for the cutoff point of dividing samples into “young” and “old” groups. Some studies compare participants under 45 years as “young” to participants between 61 and 90 years of age [10]. Grady and colleagues [14] compared young (7–45 years old) participants to the oldest old (90–109). Others divided their samples at 55 years of age [11]. We based our 45 year-old cutoff point to examine the sex differences on empirical evidence from detailed age-group analysis of the longevity-related 7-repeat allele frequencies (see Fig 1). When comparing dichotomous “young” and “old” groups with a cutoff point at 45 years we confirmed a significant increase of those carrying the 7-repeat allele (from 29.5% to 37.8%; p = 0.003), and importantly, this was preferential for females in line with the results from and colleagues. In the group of our young (18–45 years old) female participants 29.4% of the *DRD4* genotypes contained the 7 repeat allele, whereas in 46–97 aged females this ratio increased to 39.7%. This 35% significant (p = 0.003) increase closely replicates earlier findings reported by Grady and colleagues [14], who measured a 78% increase comparing a large 7–45 years old young female sample to the 90–109 year old female centenarians. Replication of both direction and gender-pecificity of accumulation of the 7 repeat allele on an ethnically different population is noteable, since even current bioinformatic specialists refer to replication as the “gold standard” for ensuring reliability of genetic-association findings [34].

Grady and colleagues [14] confirmed that carriers of the 7 repeat allele report higher levels of physical activity as compared to those lacking this genetic variant. Health benefit of exercise has been confirmed by many studies, including animal models of Alzheimer’s disease (e.g. [46]). Grady and colleagues also studied the role of the *DRD4* gene and longevity using an animal model, showing that *DRD4* gene knock-out mice lived shorter than heterozygotes or wild type mice. Furthermore, the knock-out mice showed less spontaneous locomotor activity in socially isolated, toys-deprived cages, but these effects disappeared in an enriched environment. These results suggest importance of gene environment interactions with relation to *DRD4* variants in shaping behavioral responses to environmental cues.

Obvious limitation of the present study is sample size in the detailed age groups. It should also be noted that participants of our study represent a population with high educational level and socioeconomic status, which might also influence longevity [47]. Replication studies using random samples from the general population would be important to confirm these initial findings. Investigation of other genetic variants as well as traits and behavioral characteristics of participants would permit one to draw more precise conclusions, however, it would in turn reduce overall sample size which is highly important in association studies of relatively small effects, such as this one.

Association of the *DRD4* gene variants with longevity fits well with the assumption that inheritance of longevity is complex, with modest individual genetic effects interacting with each other as well as with the environment. We propose that the *DRD4* allele 7 could be a “longevity enabling genetic variant,” protecting against basic mechanisms of age-related illnesses, but the precise manner in which this is accomplished is unclear at this point. As Grady and colleagues [14] suggest, it is likely that different *DRD4* variants might moderate longevity through specific behavioral responses to environmental cues. Our findings about the unexpected drop in 7 repeat carriers above 86 years of age might model such an interaction (see discussion above). Recently a vast amount of GWAS data was reviewed [7] related to the longitudinal New England Centenarian study with an aim to model healthy human aging. They emphasized that genetic factors might facilitate longevity through a delay or escape of age-related diseases. Identifying these factors hold a great promise for the compression of morbidity hypothesis [48], however, GWAS studies can reveal only single nucleotide variations related to longevity, and to date there are only a handful of studies on variable number of tandem repeat variations related to this topic.

In conclusion, we found that frequency of those carrying the *DRD4* 7 repeat allele continuously increased with age in a Caucasian population of Hungarians until age 75, and this was found preferentially for females. For the older groups we report a decrease in 7 repeat carriers, which might be a result of a gene environment interaction (difficult times in history might have had a selective influence on those with allele 7). All results reported here remained significant after a stringent Bonferroni correction for multiple testing.
